# Heme Oxygenase-1 in Central Nervous System Malignancies

**DOI:** 10.3390/jcm9051562

**Published:** 2020-05-21

**Authors:** Giuseppe Sferrazzo, Michelino Di Rosa, Eugenio Barone, Giovanni Li Volti, Nicolò Musso, Daniele Tibullo, Ignazio Barbagallo

**Affiliations:** 1 Department of Drug Science, Biochemistry Section, University of Catania, Viale A. Doria 6, 95125 Catania, Italy; giuseppesferrazzo95@gmail.com (G.S.); nmusso@unict.it (N.M.); 2 Department of Biomedical and Biotechnological Sciences, University of Catania, Via S. Sofia, 97 95125 Catania, Italy; chitotriosidase@gmail.com (M.D.R.); d.tibullo@unict.it (D.T.); 3 Department of Biochemical Sciences “A. Rossi-Fanelli”, Sapienza University of Rome, Piazzale A. Moro 5, 00185 Roma, Italy; eugenio.barone@uniroma1.it; 4 EuroMediterranean Institute of Science and Technology, Via Michele Miraglia 20, 90139 Palermo, Italy

**Keywords:** nuclear factor erythroid 2 p45-related factor 2, NRF2, ROS, brain cancer, oxidative stress

## Abstract

Central nervous system tumors are the most common pediatric solid tumors and account for 20–25% of all childhood malignancies. Several lines of evidence suggest that brain tumors show altered redox homeostasis that triggers the activation of various survival pathways, leading to disease progression and chemoresistance. Among these pathways, heme oxygenase-1 (HO-1) plays an important role. HO-1 catalyzes the enzymatic degradation of heme with the simultaneous release of carbon monoxide (CO), ferrous iron (Fe^2+^), and biliverdin. The biological effects of HO-1 in tumor cells have been shown to be cell-specific since, in some tumors, its upregulation promotes cell cycle arrest and cellular death, whereas, in other neoplasms, it is associated with tumor survival and progression. This review focuses on the role of HO-1 in central nervous system malignancies and the possibility of exploiting such a target to improve the outcome of well-established therapeutic regimens. Finally, several studies show that HO-1 overexpression is involved in the development and resistance of brain tumors to chemotherapy and radiotherapy, suggesting the use of HO-1 as an innovative therapeutic target to overcome drug resistance. The following keywords were used to search the literature related to this topic: nuclear factor erythroid 2 p45-related factor 2, heme oxygenase, neuroblastoma, medulloblastoma, meningioma, astrocytoma, oligodendroglioma, glioblastoma multiforme, and gliomas.

## 1. Introduction

Malignancies of the central nervous system (CNS) include neoplasia developing in the brain, spinal cord, and sellar region. Brain and other CNS tumors represent some of the most common human cancer types. In Europe, brain cancers have an incidence of 5.0 per 100,000 inhabitants/year, but no difference was evident across the various European countries [1,2]. However, in the last three decades, their incidence has increased progressively in the 65-year age group; brain cancer showed an incidence rate of 5.65 per 100,000 inhabitants in persons aged between 0–14 years [1].

Cancer cells display an altered metabolism, generally associated with an increase in reactive oxygen species (ROS) and altered redox balance resulting in cellular adaptation and proliferation [3]. Resistance to oxidative stress is one of the major adaptive advantages, allowing cancer cells to increase their metabolic rate and proliferation and to survive free radical damage. Such an adaptive response to high doses of ROS is also linked to genetic modifications, which directly or indirectly modulate ROS [4]. One of the master regulators of the antioxidant response is the nuclear factor erythroid 2 p45-related factor 2 (Nrf2) [5]. Nrf2 modulates the expression of a number of genes other than these associated with the antioxidant response and includes genes regulating immune and inflammatory responses, tissues remodeling and fibrosis, carcinogenesis, and metastasis [6].

**Figure 1:**
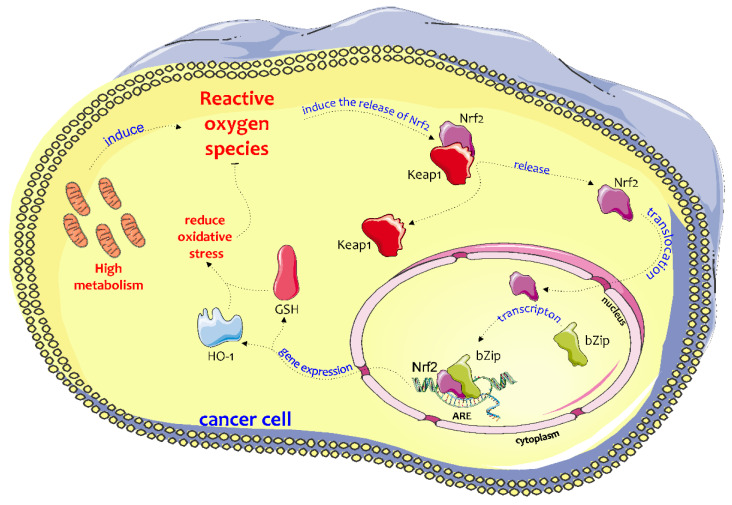
Biochemical pathways of NRF2 activation in cancer cells. Elevated metabolic rate is responsible for increased ROS production that induces Nrf2 release from Keap1 and its nuclear translocation in cancer cells. NRF2 in the nucleus binds to antioxidant response elements (ARE) and induces the expression of several antioxidant and detoxifying enzymes, such as γ-glutamate-cysteine ligase (γ-GCL), glutathione peroxidase (GPx), glutathione reductase (GR), NAD(P)H quinone dehydrogenase 1 (NQO1) and heme oxygenase-1 (HO-1). This figure was drawn using the software CorelDraw and the vector image bank of Servier Medical Art (http://smart.servier.com/). Servier Medical Art by Servier is licensed under a Creative Commons Attribution 3.0 Unported License (https://creativecommons.org/licenses/by/3.0/).

Several studies show that Nrf2 upregulation, as well as its downstream effects, are responsible for increasing cancer cell resistance to chemotherapeutic agents, such as cisplatin, doxorubicin, and etoposide [7]. In general, the biochemical mechanism through which Nrf2 confers protection or resistance (in the case of cancer cells) provides that in response to toxic stimuli, Nrf2 is released from Keap1 in the cytosol and translocates to the nucleus where Nrf2 binds to antioxidant response elements (ARE). In this way, Nrf2 induces the expression of several antioxidant and detoxifying enzymes, i.e., γ-glutamate-cysteine ligase (γ-GCL), glutathione peroxidase (GPx), glutathione reductase (GR), NAD(P)H quinone dehydrogenase 1 (NQO1) [8], and heme oxygenase-1 (HO-1) (Figure 1) [9,10,11].

HO-1 contributes to the heme degradation pathway, which involves two HO isoforms encoded by different genes [12,13]. Heme oxygenase-2 (HO-2), the constitutive isoform, is responsible for most HO activity, whilst HO-1 is induced either by its physiological substrate heme or by various stimuli including hypoxia, inflammation, and oxidative stress [14,15]. Both enzymes convert heme to equimolar amounts of ferric iron, carbon monoxide (CO) and biliverdin, which, in turn, is converted into bilirubin by biliverdin reductase [16,17]. Heme degradation involves two HO isoforms encoded by different genes, respectively [12,13]. Heme oxygenase-2 (HO-2), the constitutive isoform, is responsible for the most HO activity, whilst HO-1 is induced either by its physiological substrate, such as heme or by various stimuli including hypoxia, inflammation and oxidative stress [14,15]. Recent findings suggest that HO enzymes also possess other important ‘‘non-canonical’’ functions not related to their enzymatic activity such as protein–protein interaction, subcellular compartmentalization and secretion into the extracellular space [18], and cell metabolism [19].

HO-1 is significantly increased in various human tumors both in basal condition and during different anticancer therapies contributing, together with its by-products, to the development of a resistant phenotype [20,21,22].

In this regard, it is important to take into due account the existence of a link among Nrf2, ROS, HO-1, and p53 as the main transcription factor playing a role during cell stress response, senescence, apoptosis, and carcinogenesis [23,24]. Although p53 can have different effects depending on cell type [25], it has been reported that HO-1 stimulation in the brain is prevented in p53 null mice either under basal conditions [10,11] or under toxic stimuli including γ-irradiation [26] and hydrogen peroxide (H_2_O_2_) [27,28].

Hence, HO-1 upregulation represents an intrinsic defense to maintain cellular homeostasis and to improve cell survival. Since solid brain cancers and malignant gliomas, which occur mainly in pediatric patients, represent the greatest challenges in cancer treatment worldwide [29], deeper insight into the molecular mechanisms can help to develop novel therapeutic approaches to improve patient survival and quality of life.

The aim of the present review is to summarize the role of the HO system and its related proteins in brain cancer, also evaluating how these proteins can improve cancer prognosis and therapies. According to the latest CNS tumors classification of the World Health Organization (WHO), our study focused on embryonal tumors, meningiomas, and diffuse astrocytic and oligodendroglial tumors [30].

## 2. Biochemical Pathway of HO-1 in the Chemoresistance and Progression of Cancer

Several studies reported that HO-1 protects cancer cells and promotes their survival by conferring resistance to anticancer treatments [31,32,33]

**Figure 2:**
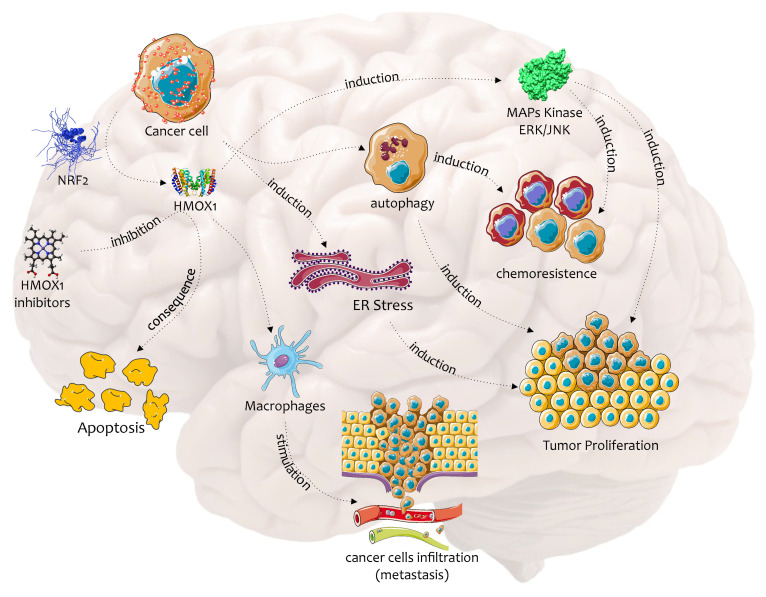
Biochemical pathway of HO-1 in the chemoresistance and progression of brain cancer. This figure was drawn using the software CorelDraw and the vector image bank of Servier Medical Art (http://smart.servier.com/). Servier Medical Art by Servier is licensed under a Creative Commons Attribution 3.0 Unported License (https://creativecommons.org/licenses/by/3.0/).

Numerous studies show that HO-1 plays a protective role in chemoresistance and tumor progression through the induction of endoplasmic reticulum stress (ER stress), autophagy, activation of MAPK kinases, and through the increase of macrophage infiltration (Figure 2) [34,35,36,37,38]. All these mechanisms offer the cell shelter from ROS damage and protein misfolding, making these cells much more resistant to damage [39]. Consistently, several studies showed that HO-1 overexpression is opposed to the therapeutic strategies implemented by proteasome inhibitors (PI), demonstrating that increased HO-1 expression limits the oxidative dysregulation responsible for misfolding of ER proteins, decreases the unfolded protein response and reduces several markers of ER stress by reducing ROS [34,40]. Other studies demonstrated that the inhibition of HO activity significantly improved the proapoptotic effect of PI and resulted in a significant reduction of the dose of PI [41,42,43].

Furthermore, Nishie et al. demonstrated that high HO-1 expression in macrophages correlates with macrophage infiltration and angiogenesis of human gliomas, suggesting that the high expression of HO-1 is associated with tumor invasiveness and poor clinical outcome [37].

Moreover, our recent study reported that HO-1 is intricate in cellular tumorigenicity involving glutathione in response to high oxidative stress triggered by the metabolism of cancer cells [44,45]. In particular, in this study, we showed that HO-1 reduces proliferation and cell viability in small lung cancer cells, inducing apoptosis mediated by glutathione resource depletion and increased oxidative.

Finally, the biological effect of HO-1 in cancer cells has been demonstrated to be cell-specific since, in some tumors, its upregulation is related to cell cycle arrest and/or death, while in other malignancies, it is associated with tumor survival and progression [46,47,48,49].

## 3. Embryonal Tumors

### 3.1. Neuroblastoma

Neuroblastoma (NB) is a pediatric solid cancer that often affects infants having an age between 0 to 4 years, with a median age of 23 months. Indeed, neuroblastoma accounts for 8% to 10% of all childhood cancers and approximately 15% of childhood cancer deaths [50]. It is a malignant embryonal tumor that occurs in the developing sympathetic nervous system derived from neural-crest tissues cells [51]. Neuroblastic tumors, according to neuronal differentiation and Schwannian stroma content, could be classified as ganglioneuromas (GNs), composed of mature ganglion cells, ganglioneuroblastomas (GNBs), composed of mature ganglion cells and neuroblasts, and NBs, composed of neuroblasts. NBs are, in turn, classified into differentiated, poorly differentiated, and undifferentiated tumors [52]. NBs present mass lesions in the neck, chest, abdomen, or pelvis and generally have a fatal prognosis [53,54].

Several factors, such as age and disease stage at diagnosis, as well as genetic features of the tumor, could determine the patient’s final outcome, ranging from spontaneous regression to metastatic neoplasms. Indeed, the ploidy status, MYCN oncogene amplification, or allelic loss in chromosome 1p, correlate with different clinical phenotypes [53,55]. Notably, MYCN amplification—the powerful adverse prognostic factor occurring in 16% to 25% of NB patients—causes chemotherapy resistance, especially in advanced stage neuroblastomas [56].

Several lines of evidence propose proteasome inhibitors (PIs) as chemotherapic agents for solid tumors, including NB [57]. In fact, the inhibition of proteasome activity increases the apoptotic rate and sensitizes tumor cells to traditional chemo- and radiotherapies [58,59].

A study conducted by Furfaro et al. reported that NRF2 and HO-1 upregulation decrease the efficacy of bortezomib (BTZ)—the first selective and reversible 26S PI tested in clinical trials [60]—in treating NB cells. Conversely, the inhibition of HO-1, obtained with Zn protoporphyrin IX (ZnPPIX), improved the proapoptotic effect of BTZ, resulting in a significant reduction of the PI dose and preventing NB cell resistance to BTZ [41]. Furthermore, they also showed that the synergic effect of HO-1 silencing and glutathione depletion, obtained with L-buthionine-(S,R)-sulphoximine (BSO), significantly decrease the viability of BTZ-treated HTLA-230 NB cells [61].

Similarly, in GI-ME-N cell resistance to the chemotherapic etoposide, the Nrf2/HO-1 pathway plays a crucial role as an adaptive response to cellular stress induced from GSH depletion. Indeed, authors showed that NRF2 and HO-1 silencing sensitizes GI-ME-N cells to BSO stress and then to etoposide [62].

In line with these results, our study demonstrated that the silencing of HO-1 with a novel non-competitive inhibitor (LS1/71) makes SH-SY5Y NB cells more sensitive to carfilzomib (CFZ). Interestingly, CFZ treatment also induces the ERK and JNK signal transduction pathways, promoting cell proliferation and decreasing apoptosis rate. By contrast, LS1/71 was able to counteract these effects inhibiting ERK and JNK phosphorylation [42].

Furthermore, several studies demonstrated that miR-494, involved in HO-1 induction and cell responsive to oxidative stress, is expressed in NB cells [63]. These studies show that after differentiation with all-trans retinoic acid (ATRA), the expression levels of miR-494 and HO-1 are significantly downregulated in SH-SY5Y and SK-N-BE cell lines, reducing their viability after H_2_O_2_ treatment. In particular, cell sensitivity to oxidative stress was increased by the impairment of Bach1-dependent HO-1. Since the HO-1 promoter is hidden by Bach1 in ATRA differentiated cells, Nrf2 is unable to match with the HO-1 promoter [64,65].

Fest et al. tested the effect of HO-1 inhibition on NB progression in an in vivo model using A/J mice (H2-K^K^), which were treated with a sublethal subcutaneous dose of NXS2 cells and then ZnPPIX or sodium chloride were administered before surgery. HO-1 inhibition significantly reduced tumor growth, volume and liver metastasis, and induced apoptosis, decreasing Bcl2 and Bcl-Xl levels. Moreover, HO-1 inhibition stimulated immune cells to attack tumors promoting NXS2 cell lyse [66].

Conversely, several studies proposed HO-1 overexpression as a potential treatment for NB. In particular, Hassannia et al. investigated the effect of the natural anticancer withaferin A (WA) in inducing a non-canonical ferroptosis mediated by an abnormal HO-1 overexpression in IMR-32 and SK-N-SH NB cells. Targeting Kelch-like ECH-associated protein 1 (KEAP1), WA was capable of increasing Fe(II) levels through HO-1 overexpression [67].

**Figure 3:**
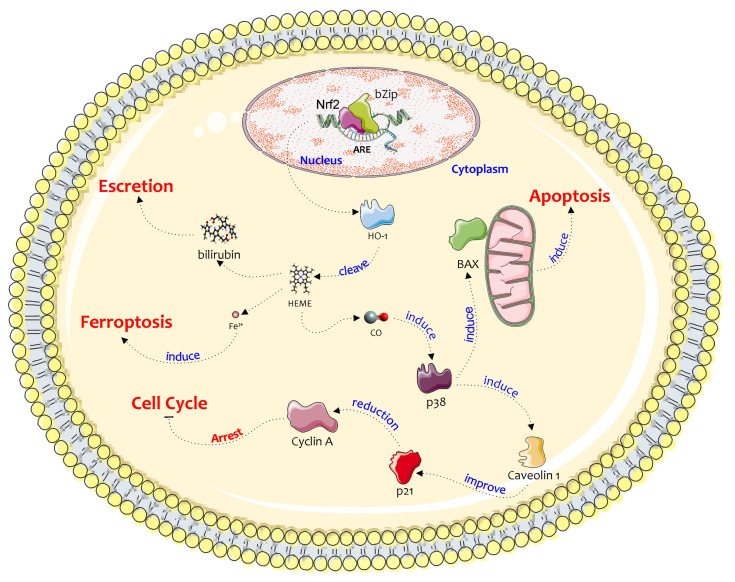
A possible anticancer pathway of HO-1 in neuroblastoma cells. The figure shows different pathways induced by NRF2 action on HO-1. All pathways converge through the stress of other factors, such as the splitting of the heme group and the release of Fe + ions, the arrest of the cell cycle with induction of p21 or through BAX and classic apoptosis. This figure was drawn using the software CorelDraw and the vector image bank of Servier Medical Art (http://smart.servier.com/). Servier Medical Art by Servier is licensed under a Creative Commons Attribution 3.0 Unported License (https://creativecommons.org/licenses/by/3.0/).

Accordingly, Hayama et al. demonstrated that through HO-1 induction, ferrearin-type neolignans cause apoptotic activity in human IMR-32, LA-N-1, NB-39, and SK-N- SH cell lines [68]. This effect is related to the CO produced by HO-1 that stimulates p38-MAPK and the JNK pathway, inducing BAX overexpression and apoptosis [69] (Figure 3).

Furthermore, p38-MAPK activation leads to a significantly increased expression of caveolin-1, which in turn leads to the expression of p21, the reduction of cyclin A and thus to cell growth arrest (Figure 2) [70]. Similar results were obtained by Kitano et al. using a vitamin K analog, VK3-OCH3, which caused a G2/M cell cycle arrest and apoptosis through the above-mentioned HO-1 signal transduction pathway [71].

In conclusion, although several studies have proposed HO-1 as a possible marker of chemoresistance, the aforementioned studies highlighted the contradictory effects of HO-1 expression in NB cell survival.

### 3.2. Medulloblastoma

Medulloblastoma is the most malignant cerebellar tumor, accounting for approximately 10% of all childhood brain tumors. It is characterized by a significant burden of adverse outcomes in survivors [72]. According to pathological features, medulloblastoma is classified into classic, desmoplastic/nodular, extensively nodular, large cell, and anaplastic. In particular, the last two variants lead to a worse prognosis and metastatic diseases [72,73]. Furthermore, based on a unique genetic pathway and common clinical characteristics, medulloblastomas are categorized in wingless (WNT), sonic hedgehog (SHH), group 3 (high levels of MYC expression) and group 4. Traditional genetic analysis suggests that isochromosome 17q, which results in the loss of 17p and the gain of 17q, represents the most common aberration in medulloblastomas [74,75,76].

Therapeutic strategies for medulloblastoma include a combination of surgery, radiotherapy, and chemotherapy [75,77]. Since medulloblastoma is a chemosensitive tumor, children affected are generally treated with methotrexate, and high-dose cyclophosphamide, cisplatinum, and carboplatinum in children with recurrent medulloblastoma. Recent studies have reported that the use of combined chemotherapy, such as ifosfamide, carboplatinum, and etoposide, has shown remarkable results [78,79]. Notably, Li et al. evaluated the role of HO-1and HO-2 on different clinical and pathological characteristics of medulloblastoma, but no significant difference was found with the different tumor subtypes. Although HO-1 and HO-2 expression also showed no significant association with the different medulloblastoma subtypes, patients with high HO-1 and low HO-2 expression have better survival [80].

Recent findings examined the capacity of HO-1 induction of decreasing DAOY medulloblastoma cell sensitivity to oxidant-induced death. With this in mind, HO-1 induction played a cytoprotective role against oxidative stress in DAOY cells exposed to the CO donor. The capacity of endogenous CO production of preventing apoptosis may be responsible for medulloblastomas’ resistance to therapy. These results suggest that pharmacological targeting of HO-1 could enhance the efficacy of radiotherapy or chemotherapy [81,82].

### 3.3. Meningiomas

Meningioma is a common tumor of the central nervous system affecting the meninges. Generally, it could be originated by any intracranial, intraventricular, or spinal dural surface. In accordance with the WHO 2016 guidelines, meningiomas are classified as Grade I, Grade II or “atypical”, and Grade III or “anaplastic” [83,84]. Genetic alterations, such as mutation or loss of the tumor suppressor gene neurofibromatosis 2 (NF2) on chromosome 22, constitute a leading cause of about 50% of meningiomas [85,86].

Recent studies reported that non-NF2 meningiomas are determined by the following mutations, including SMO, SUFU, PRKAR1A, KLF4, PIK3CA, POLR2A, and other rarer mutations [87,88,89].

Current guidelines suggested surgery followed by radiotherapy as treatment of choice for intracranial meningiomas. Indeed, chemotherapy is still considered a field under investigation in the context of meningiomas. Indeed, new inhibitors, such as vistusertib, nivolumab, and pembrolizumab, are being tested in clinical trials [90,91].

A study conducted by Takahashi et al. showed that HO-1 induction in rat KMY-J cells treated with TS-PDT (photodynamic therapy using talaporfin sodium) may contribute to resistance in meningioma cells, also attenuating its therapeutic effect.

In particular, the mRNA expression level of Hmox1 was significantly increased, and this effect was counteracted when TS-PDT was combined with ZnPPIX, reducing meningioma cell viability [92].

## 4. Diffuse Astrocytic and Oligodendroglial Tumors

Gliomas, the most common group of primary brain tumors, are classified by the WHO into four grades, based on histological characteristics, with different prognosis and survival. In particular, gliomas include astrocytoma, oligodendroglioma, ependymoma and glioblastoma multiforme [30].

### 4.1. Astocytoma

Astrocytomas are most frequently caused by several chromosomal alterations, such as trisomy or polysomy of chromosome 7. Indeed, recent studies using comparative genomic hybridization found this abnormality in more than 50% of cases [93,94]. Moreover, losses of 22q, 19q,13q, 11p, 10p, 9p, and 6, as well as the sex chromosomes or the mutations in TP53, CDKN2A, p14ARF, and CDKN2B tumor suppressor genes, also result in a small percentage of diffuse and anaplastic astrocytomas [95,96]. Astrocytomas, especially those showing LOH on 17p, are frequently characterized by a higher expression of the platelet-derived growth factor receptor A (PDGFRA) and its ligand PDGFa, suggesting an autocrine growth stimulation [97,98].

Several lines of evidence suggest a marked association between HO-1 expression levels and brain tumors compared to normal brains [99,100]. Interestingly, the HO-1 level was higher in all tumor histological subtypes, but no differences were observed with the various tumor grades. In particular, HO-1 expression was involved in a worse prognosis of patients with Grades II and III astrocytomas, suggesting a pro-tumoral role of HO-1 in glioma progression [101].

### 4.2. Oligodendroglioma

Oligodendroglial tumors comprise oligodendrogliomas and oligoastrocytomas, accounting for less than 10% of the diffuse gliomas [102,103]. Frequently, oligodendroglial tumors are caused by the loss of heterozygosity (LOH) for chromosome arms 1p and 19q, which, in turn, derive from a non-balanced translocation t (1:19) (q10:p10) [104]. However, adult oligodendroglioma shows the 1p19q codeletion pattern; it may include 1p loss without 19q deletion [105]. Moreover, diffuse gliomas of Grades II and III present a high percentage of IDH1 and IDH2 mutations [106].

In high-grade gliomas, HO-1 expression correlates with macrophage infiltration and vascular density, contributing to neoplastic growth and necrosis. Indeed, HO-1-expressing macrophages are higher in areas of solid tumor growth and decrease with increasing tumor distance. HO-1 accumulation is also proposed as an indicator of neoangiogenesis in hypoxic areas [37,99]. Furthermore, HO-mediated heme degradation is involved in cellular CO production, which is capable of inducing angiogenesis and neoplastic growth [107].

### 4.3. Glioblastoma Multiforme

Glioblastoma multiforme (GBM) is the most aggressive glioma grade characterized by highly cellular proliferation, diffuse infiltration, marked angiogenesis, resistance to apoptosis, and genomic instability. Furthermore, GBM has been divided into primary and secondary subgroups according to clinical characteristics [108].

Malignant transformation is caused by genetic alterations and dysregulations of different growth factor signaling pathways such as vascular endothelial growth factor (VEGF), epidermal growth factor (EGF), platelet-derived growth factor (PDGF), and phosphatase and tensin homolog (PTEN) [109]. Loss of heterozygosity (LOH) (i.e., on chromosome 10) is one of the most frequent genetic alterations across the genome [110,111]. Moreover, the most common triggering events for glioblastoma formation are amplifications of the EGFR gene on chromosome 7, or mutations in the TP53 or the retinoblastoma (RB1) pathways [112,113,114].

Currently, patients younger than 70 years old receive surgical intervention combined with radiotherapy and chemotherapy. Several studies highlighted that Nrf2 influences the proliferation of GSCs, also inducing the relapse and invasion of the tumors. Both in vitro and in vivo studies have reported the role of Nrf2 in blocking the proliferation of human glioma, confirming the contribution of Nrf2 in maintaining self-renewal in GSCs [115].

Interestingly, Sun et al. showed that the inhibition of NRF2 expression during ferroptosis-targeted therapies is a key factor in enhancing therapeutic tumor effects [116]. Therefore, Nrf2 inhibition could induce GSC differentiating into non-stem-like glioblastoma cells, reducing tumor growth, and increasing the sensitivity to radiotherapy and chemotherapy [117].

The proangiogenic factors HO-1, VEGF, and basic FGF (bFGF) upregulate metastatic activities in various tumor systems [118]. Thus, HO-1 could be considered a potential target to counteract both initial and metastatic grade tumor growth [119]. In fact, when HO-1 is not expressed, the GBM cell invasion is inhibited [120].

The protein kinases—extracellular-signal-regulated kinases (ERK), phosphatidylinositol 3-kinases (PI3K), protein kinase C (PKC), and glycogen synthase kinase 3-β (GSK3-β)—play a pivotal role in Nrf2 regulation [121]. Cong et al. demonstrated that the combined inhibition of ERK and PI3K signaling decreased the expression and activation of Nrf2, also suppressing glioma cell viability partially through Nrf2-ARE downstream target genes [122].

Moreover, Pan et al. revealed that upregulated expression of Nrf2 reduced apoptosis in glioma cell line U251 and enhanced expression of HO-1; on the other hand, downregulation of Nrf2 increased apoptosis and reduced expression of HO-1. Summarizing briefly, HO-1 changed according to Nrf2, indicating that HO-1 may participate in apoptosis regulated by Nrf2 in U251 [123]. Furthermore, U251 cells treated with ZnPPIX augmented the anticancer effects of arsenic trioxide (ATO), inhibiting NRF2 and HO-1, while these effects were counteracted in cells treated with cobalt protoporphyrin (CoPPIX), a well-known HO-1 inducer [124].

Accordingly, Zhang et al. demonstrated the role of FTY720 as a potent inhibitor of NFR2 in U251MG and U87MG GBM cell lines. Indeed, FTY720 was able to downregulate NFR2 expression increasing susceptibility of GBM cells to the cytotoxic effect of TMZ treatment, while NRF2 induction had an opposite effect [125].

Since HO-1 gene expression correlates with neovascularization, it is considered a potential marker of macrophage infiltration as well as neovascularization in human gliomas. As showed by Andaloussi et colleagues, the expression of HO-1 was directly correlated with FoxP3, a marker of regulatory T-cells (Treg), and tumor growth. Indeed, Treg progressively infiltrates gliomas with an increase in brain tumor grade, determining an immunosuppressive environment [37,126]. Furthermore, GBM cells display a hypoxia-dependent differential modulation of biliverdin reductase (hBVR), increasing its expression and promoting cell survival under hypoxic states. In particular, hypoxic conditions determine a significant increase in hBVR expression in GBM cells, which is accompanied by chemoresistance. Conversely, the inhibition of hBVR activity could overcome hypoxia-induced chemoresistance by regulating the cellular redox grade [127].

Recent studies have evaluated the role of HO-1 upregulation in the regulation of ferroptosis in cancer therapeutics, mainly in chemoresistance, small molecule-induced ferroptosis inhibits tumor progression and improves the sensitivity of chemotherapeutic drugs. Increased expression of HO-1 is crucial in erastin-induced ferroptosis in HT-1080 fibrosarcoma cells, also providing iron supplements for stimulating ferroptosis [128,129].

Kyani et al. described pyrimidotriazinedione 35G8, a protein disulfide isomerase (PDI) inhibitor, as toxic for human glioblastoma cell lines. Interestingly, 35G8 upregulated heme oxygenase 1 and SLC7A11 (solute carrier family 7 member 11) and inhibited PDI target genes such as TXNIP (thioredoxin-interacting protein 1) and EGR1 (early growth response 1). Moreover, 35G8-induced cell death derived from a combination of autophagy and ferroptosis, a death form independent of apoptosis and necrosis [130,131].

In conclusion, although several studies proposed Nrf2 and HO-1 inhibition as a possible strategy to sensitize resistant glioma cells to chemotherapy, the above-mentioned studies demonstrated a non- univocal role of HO-1 expression.

## 5. HO-1 Inhibitors and Their Potential Use in the Treatment of CNS Malignancies

Many studies report the positive effects of HO-1 inhibitors on many types of cancers. These promising ways of inhibition of HO-1 are founded on the genetic inhibition of HMOX1 by silencing RNA, on the use of metalloporphyrins (zinc protoporphyrin-ZnPPIX, tin protoporphyrin -SnPPIX, or chromium protoporphyrin-CrPPIX) and on the use of imidazole-based compounds [132]. However, many of the inhibitors used in medical research are part of the category of metalloporphyrins that, in addition to having a competitive interaction with HO-1, are non-specific because they also inhibit HO-2, the constitutive isoform. Moreover, imidazole-based compounds are characterized by a non-competitive binding mode showing high selectivity enzymatic activity inhibition of HO-1 with respect to HO-2 [133,134].

Many of imidazole-based compounds were tested in in vitro studies of cancer models such as neuroblastoma, myeloma, melanoma, and lung cancer [42,44,135,136,137]. These inhibitors, also used in combination with other chemotherapeutic drugs, have shown excellent results by inducing apoptosis or cell cycle arrest in the tested models [42,49,138].

## 6. Conclusions

Taken together, the aforementioned studies suggest the HO system plays a crucial role in chemoresistance and progression of brain cancer. Particularly, these results show a strong association between HO-1 upregulation and both progression and drug-resistance in brain tumors, suggesting HO-1 system modulation to improve chemotherapy sensitivity.

The examined studies show that the cytoprotective and antioxidant effect of HO1 is responsible for the increased progressivity and malignancy of tumors, inducing malignant cell adaptation to high cellular oxidative stress stimulated by high metabolism or chemotherapy (Figure 2).

Interestingly, some studies highlight the opposite effects of HO-1 expression, presuming that these are not dependent on the HO-1 system alone (Figure 3). In the findings analyzed in which HO-1 overexpression resolved in a cell division block or in a trigger of apoptosis, the observed effects were related to the increase in cytotoxicity caused by the by-products of heme degradation. In particular, the increase in iron concentration was able to produce ferroptosis.

In Table 1, we report a list of CNS tumors in which upregulation or downregulation of HO-1 is associated with an arrest in cell cycle division and subsequent cellular death or tumor survival and progression.

In order to confirm HO-1 as a possible molecular target for brain cancer, further research should be performed considering several limitations such as paucity of in vivo studies, lack of information about the role of the HO-1 system in the glioma cell environment, the non-canonical functions of HO-1, and the epigenetic mechanisms on HO-1 gene expression. In conclusion, understanding the mechanisms related to the HO-1 system may offer an additional target for future therapies and ameliorate oncological patients’ outcomes.

## Figures and Tables

**Table 1:** List of studies carried out in CNS tumors in which HO-1 expression was analyzed. The table shows tissues or cell lines, HO-1 expression, treatments, and outcomes with relevant references.

Tumor	Cell line	HO-1 Expression	Treatment	Outcome	Reference
**NB**	HTLA-230	↓	BTZ	Apoptosis	[61,62]
**NB**	GI-ME-M	↓	Etoposide	Apoptosis	[63]
**NB**	SH-SY5Y	↓	CFZ	Apoptosis	[42]
**NB**	SH-SY5Y; SK-N-BE	↓	H_2_O_2_	↓ Viability	[64,65]
**NB**	A/J Mice (H_2_-K^k^)	↓	NXS2	↓ Tumor growth, volume, and metastasis	[66]
**NB**	IMR-32; SK-H-SH	↑↑↑	WA	Ferroptosis	[67]
**NB**	IMR-32; LA-N-1; NB-39; SK-N-SH	↑	Ferrearin-type neolignans	Apoptosis	[68]
**NB**	IMR-32; LA-N-1	↑	VK3-OCH_3_	G2/M cell cycle arrest, apoptosis	[71]
**MB**	Resected specimens	↑		Protect tumor cells	[80]
**MB**	DAOY	↑	ROS	↑ Viability	[81]
**MG**	KMY-J	↓	TS-PDT	↓ Viability	[92]
**ASTRO**	Sample from Biorepository	↑		Worse prognosis	[101]
**OD**	rat intracranially transplanted C6 gliomas and Resected specimens	↑		Macrophage infiltration, tumor growth and angiogenesis	[37,99]
**GBM**	Primary GBM cell	↓		Inhibits GBM cell invasion	[120]
**GBM**	U251	↓		Apoptosis	[123]
**GBM**	U251	↓	ATO	Apoptosis	[124]
**GBM**	Resected specimens	↑		↑ Treg infiltration	[126]
**GBM**	U87MG	↑	35G8	Autophagy and ferroptosis	[131]

## References

[1] Ostrom Q.T., Gittleman H., Truitt G., Boscia A., Kruchko C., Barnholtz-Sloan J.S. (2018). CBTRUS Statistical Report: Primary Brain and Other Central Nervous System Tumors Diagnosed in the United States in 2011–2015. Neuro Oncol..

[2] Jensen O.M., Estève J., Møller H., Renard H. (1990). Cancer in the European Community and its member states. Eur. J. Cancer.

[3] DeBerardinis R.J., Chandel N.S. (2016). Fundamentals of cancer metabolism. Sci. Adv..

[4] Reuter S., Gupta S.C., Chaturvedi M.M., Aggarwal B.B. (2010). Oxidative stress, inflammation, and cancer: How are they linked?. Free Radic. Biol. Med..

[5] Kensler T.W., Wakabayashi N., Biswal S. (2007). Cell survival responses to environmental stresses via the Keap1-Nrf2-ARE pathway. Annu. Rev. Pharmacol. Toxicol..

[6] Hybertson B.M., Gao B., Bose S.K., McCord J.M. (2011). Oxidative stress in health and disease: The therapeutic potential of Nrf2 activation. Mol. Aspects Med..

[7] Wang X.J., Sun Z., Villeneuve N.F., Zhang S., Zhao F., Li Y., Chen W., Yi X., Zheng W., Wondrak G.T. (2008). Nrf2 enhances resistance of cancer cells to chemotherapeutic drugs, the dark side of Nrf2. Carcinogenesis.

[8] Uruno A., Motohashi H. (2011). The Keap1-Nrf2 system as an in vivo sensor for electrophiles. Nitric Oxide.

[9] Kwak M.K., Wakabayashi N., Kensler T.W. (2004). Chemoprevention through the Keap1-Nrf2 signaling pathway by phase 2 enzyme inducers. Mutat. Res..

[10] Barone E., Cenini G., Sultana R., Di Domenico F., Fiorini A., Perluigi M., Noel T., Wang C., Mancuso C., St Clair D.K. (2012). Lack of p53 decreases basal oxidative stress levels in the brain through upregulation of thioredoxin-1, biliverdin reductase-A, manganese superoxide dismutase, and nuclear factor kappa-B. Antioxid. Redox Signal..

[11] Barone E., Cenini G., Di Domenico F., Noel T., Wang C., Perluigi M., St Clair D.K., Butterfield D.A. (2015). Basal brain oxidative and nitrative stress levels are finely regulated by the interplay between superoxide dismutase 2 and p53. J. Neurosci. Res..

[12] Shibahara S., Yoshizawa M., Suzuki H., Takeda K., Meguro K., Endo K. (1993). Functional analysis of cDNAs for two types of human heme oxygenase and evidence for their separate regulation. J. Biochem..

[13] McCoubrey W.K., Ewing J.F., Maines M.D. (1992). Human heme oxygenase-2: Characterization and expression of a full-length cDNA and evidence suggesting that the two HO-2 transcripts may differ by choice of polyadenylation signal. Arch. Biochem. Biophys..

[14] Ryter S.W., Alam J., Choi A.M. (2006). Heme oxygenase-1/carbon monoxide: From basic science to therapeutic applications. Physiol. Rev..

[15] Kushida T., LiVolti G., Goodman A.I., Abraham N.G. (2002). TNF-alpha-mediated cell death is attenuated by retrovirus delivery of human heme oxygenase-1 gene into human microvessel endothelial cells. Transplant. Proc..

[16] Tenhunen R., Marver H.S., Schmid R. (1968). The enzymatic conversion of heme to bilirubin by microsomal heme oxygenase. Proc. Natl. Acad. Sci. USA.

[17] Barone E., Di Domenico F., Mancuso C., Butterfield D.A. (2014). The Janus face of the heme oxygenase/biliverdin reductase system in Alzheimer disease: It’s time for reconciliation. Neurobiol. Dis..

[18] Vanella L., Barbagallo I., Tibullo D., Forte S., Zappala A., Li Volti G. (2016). The non-canonical functions of the heme oxygenases. Oncotarget.

[19] Barone E., Butterfield D.A. (2015). Insulin resistance in Alzheimer disease: Is heme oxygenase-1 an Achille’s heel?. Neurobiol. Dis..

[20] Goodman A.I., Choudhury M., da Silva J.L., Schwartzman M.L., Abraham N.G. (1997). Overexpression of the heme oxygenase gene in renal cell carcinoma. Proc. Soc. Exp. Biol. Med..

[21] Berberat P.O., Dambrauskas Z., Gulbinas A., Giese T., Giese N., Kunzli B., Autschbach F., Meuer S., Buchler M.W., Friess H. (2005). Inhibition of heme oxygenase-1 increases responsiveness of pancreatic cancer cells to anticancer treatment. Clin. Cancer Res. Off. J. Am. Assoc. Cancer Res..

[22] Barbagallo I., Parenti R., Zappalà A., Vanella L., Tibullo D., Pepe F., Onni T., Li Volti G. (2015). Combined inhibition of Hsp90 and heme oxygenase-1 induces apoptosis and endoplasmic reticulum stress in melanoma. Acta Histochem..

[23] Mijit M., Caracciolo V., Melillo A., Amicarelli F., Giordano A. (2020). Role of p53 in the Regulation of Cellular Senescence. Biomolecules.

[24] Beyfuss K., Hood D.A. (2018). A systematic review of p53 regulation of oxidative stress in skeletal muscle. Redox Rep..

[25] Lozano G. (2010). Mouse models of p53 functions. Cold Spring Harb. Perspect. Biol..

[26] Meiller A., Alvarez S., Drane P., Lallemand C., Blanchard B., Tovey M., May E. (2007). p53-dependent stimulation of redox-related genes in the lymphoid organs of gamma-irradiated--mice identification of Haeme-oxygenase 1 as a direct p53 target gene. Nucleic Acids Res..

[27] Nam S.Y., Sabapathy K. (2011). p53 promotes cellular survival in a context-dependent manner by directly inducing the expression of haeme-oxygenase-1. Oncogene.

[28] Mauri E., Sacchetti A., Vicario N., Peruzzotti-Jametti L., Rossi F., Pluchino S. (2018). Evaluation of RGD functionalization in hybrid hydrogels as 3D neural stem cell culture systems. Biomater. Sci..

[29] Izycka-Swieszewska E., Bien E., Stefanowicz J., Szurowska E., Szutowicz-Zielinska E., Koczkowska M., Sigorski D., Kloc W., Rogowski W., Adamkiewicz-Drozynska E. (2018). Malignant Gliomas as Second Neoplasms in Pediatric Cancer Survivors: Neuropathological Study. Biomed. Res. Int..

[30] Louis D.N., Perry A., Reifenberger G., von Deimling A., Figarella-Branger D., Cavenee W.K., Ohgaki H., Wiestler O.D., Kleihues P., Ellison D.W. (2016). The 2016 World Health Organization Classification of Tumors of the Central Nervous System: A summary. Acta Neuropathol..

[31] Ryter S.W., Choi A.M. (2009). Heme oxygenase-1/carbon monoxide: From metabolism to molecular therapy. Am. J. Respir. Cell Mol. Biol..

[32] Jozkowicz A., Was H., Dulak J. (2007). Heme oxygenase-1 in tumors: Is it a false friend?. Antioxid. Redox Signal..

[33] Di Mauro R., Cantarella G., Bernardini R., Di Rosa M., Barbagallo I., Distefano A., Longhitano L., Vicario N., Nicolosi D., Lazzarino G. (2019). The Biochemical and Pharmacological Properties of Ozone: The Smell of Protection in Acute and Chronic Diseases. Int. J. Mol. Sci..

[34] Lee G.H., Kim H.K., Chae S.W., Kim D.S., Ha K.C., Cuddy M., Kress C., Reed J.C., Kim H.R., Chae H.J. (2007). Bax inhibitor-1 regulates endoplasmic reticulum stress-associated reactive oxygen species and heme oxygenase-1 expression. J. Biol. Chem..

[35] Vasconcellos L.R., Siqueira M.S., Moraes R., Carneiro L.A., Bozza M.T., Travassos L.H. (2018). Heme Oxygenase-1 and Autophagy Linked for Cytoprotection. Curr. Pharm. Des..

[36] Pei L., Kong Y., Shao C., Yue X., Wang Z., Zhang N. (2018). Heme oxygenase-1 induction mediates chemoresistance of breast cancer cells to pharmorubicin by promoting autophagy via PI3K/Akt pathway. J. Cell. Mol. Med..

[37] Nishie A., Ono M., Shono T., Fukushi J., Otsubo M., Onoue H., Ito Y., Inamura T., Ikezaki K., Fukui M. (1999). Macrophage infiltration and heme oxygenase-1 expression correlate with angiogenesis in human gliomas. Clin. Cancer Res..

[38] So K.Y., Kim S.H., Jung K.T., Lee H.Y., Oh S.H. (2017). MAPK/JNK1 activation protects cells against cadmium-induced autophagic cell death via differential regulation of catalase and heme oxygenase-1 in oral cancer cells. Toxicol. Appl. Pharmacol..

[39] Johnson D.E. (2015). The ubiquitin-proteasome system: Opportunities for therapeutic intervention in solid tumors. Endocr. Relat. Cancer.

[40] Kim Y., Li E., Park S. (2012). Insulin-like growth factor-1 inhibits 6-hydroxydopamine-mediated endoplasmic reticulum stress-induced apoptosis via regulation of heme oxygenase-1 and Nrf2 expression in PC12 cells. Int. J. Neurosci..

[41] Furfaro A.L., Piras S., Passalacqua M., Domenicotti C., Parodi A., Fenoglio D., Pronzato M.A., Marinari U.M., Moretta L., Traverso N. (2014). HO-1 up-regulation: A key point in high-risk neuroblastoma resistance to bortezomib. Biochim. Biophys. Acta.

[42] Barbagallo I., Giallongo C., Volti G.L., Distefano A., Camiolo G., Raffaele M., Salerno L., Pittala V., Sorrenti V., Avola R. (2019). Heme Oxygenase Inhibition Sensitizes Neuroblastoma Cells to Carfilzomib. Mol. Neurobiol..

[43] Gulino R., Vicario N., Giunta M.A.S., Spoto G., Calabrese G., Vecchio M., Gulisano M., Leanza G., Parenti R. (2019). Neuromuscular Plasticity in a Mouse Neurotoxic Model of Spinal Motoneuronal Loss. Int. J. Mol. Sci..

[44] Spampinato M., Sferrazzo G., Pittala V., Di Rosa M., Vanella L., Salerno L., Sorrenti V., Carota G., Parrinello N., Raffaele M. (2020). Non-competitive heme oxygenase-1 activity inhibitor reduces non-small cell lung cancer glutathione content and regulates cell proliferation. Mol. Biol. Rep..

[45] Vicario N., Bernstock J.D., Spitale F.M., Giallongo C., Giunta M.A.S., Li Volti G., Gulisano M., Leanza G., Tibullo D., Parenti R. (2019). Clobetasol Modulates Adult Neural Stem Cell Growth via Canonical Hedgehog Pathway Activation. Int. J. Mol. Sci..

[46] Becker J.C., Fukui H., Imai Y., Sekikawa A., Kimura T., Yamagishi H., Yoshitake N., Pohle T., Domschke W., Fujimori T. (2007). Colonic expression of heme oxygenase-1 is associated with a better long-term survival in patients with colorectal cancer. Scand. J. Gastroenterol..

[47] Li Volti G., Tibullo D., Vanella L., Giallongo C., Di Raimondo F., Forte S., Di Rosa M., Signorelli S.S., Barbagallo I. (2017). The Heme Oxygenase System in Hematological Malignancies. Antioxid. Redox Signal..

[48] Nitti M., Piras S., Marinari U.M., Moretta L., Pronzato M.A., Furfaro A.L. (2017). HO-1 Induction in Cancer Progression: A Matter of Cell Adaptation. Antioxidants.

[49] Salerno L., Romeo G., Modica M.N., Amata E., Sorrenti V., Barbagallo I., Pittala V. (2017). Heme oxygenase-1: A new druggable target in the management of chronic and acute myeloid leukemia. Eur. J. Med. Chem..

[50] Park J.R., Eggert A., Caron H. (2010). Neuroblastoma: Biology, prognosis, and treatment. Hematol. Oncol. Clin. N. Am..

[51] Hoehner J.C., Gestblom C., Hedborg F., Sandstedt B., Olsen L., Påhlman S. (1996). A developmental model of neuroblastoma: Differentiating stroma-poor tumors’ progress along an extra-adrenal chromaffin lineage. Lab. Investig..

[52] Lonergan G.J., Schwab C.M., Suarez E.S., Carlson C.L. (2002). Neuroblastoma, ganglioneuroblastoma, and ganglioneuroma: Radiologic-pathologic correlation. Radiographics.

[53] Maris J.M. (2010). Recent advances in neuroblastoma. N. Engl. J. Med..

[54] Castel Sánchez V., Melero Moreno C., García-Miguel García-Rosados P., Navajas Gutiérrez A., Ruiz Jiménez J.I., Navarro Fos S., Garín Valle J.C., Galbe Sada M. (1997). Neuroblastoma in children under than 1 year of age. An. Esp. Pediatr..

[55] Brodeur G.M. (2003). Neuroblastoma: Biological insights into a clinical enigma. Nat. Rev. Cancer.

[56] Cohn S.L., Pearson A.D., London W.B., Monclair T., Ambros P.F., Brodeur G.M., Faldum A., Hero B., Iehara T., Machin D. (2009). The International Neuroblastoma Risk Group (INRG) classification system: An INRG Task Force report. J. Clin. Oncol..

[57] Mody R., Zhao L., Yanik G.A., Opipari V. (2017). Phase I study of bortezomib in combination with irinotecan in patients with relapsed/refractory high-risk neuroblastoma. Pediatric Blood Cancer.

[58] Voorhees P.M., Dees E.C., O’Neil B., Orlowski R.Z. (2003). The proteasome as a target for cancer therapy. Clin. Cancer Res..

[59] Joazeiro C.A., Anderson K.C., Hunter T. (2006). Proteasome inhibitor drugs on the rise. Cancer Res..

[60] Adams J., Kauffman M. (2004). Development of the proteasome inhibitor Velcade (Bortezomib). Cancer Investig..

[61] Furfaro A.L., Piras S., Domenicotti C., Fenoglio D., De Luigi A., Salmona M., Moretta L., Marinari U.M., Pronzato M.A., Traverso N. (2016). Role of Nrf2, HO-1 and GSH in Neuroblastoma Cell Resistance to Bortezomib. PLoS ONE.

[62] Furfaro A.L., Macay J.R., Marengo B., Nitti M., Parodi A., Fenoglio D., Marinari U.M., Pronzato M.A., Domenicotti C., Traverso N. (2012). Resistance of neuroblastoma GI-ME-N cell line to glutathione depletion involves Nrf2 and heme oxygenase-1. Free Radic. Biol. Med..

[63] Piras S., Furfaro A.L., Caggiano R., Brondolo L., Garibaldi S., Ivaldo C., Marinari U.M., Pronzato M.A., Faraonio R., Nitti M. (2018). microRNA-494 Favors HO-1 Expression in Neuroblastoma Cells Exposed to Oxidative Stress in a Bach1-Independent Way. Front. Oncol..

[64] Yao P.L., Chen L., Dobrzański T.P., Zhu B., Kang B.H., Müller R., Gonzalez F.J., Peters J.M. (2017). Peroxisome proliferator-activated receptor-β/δ inhibits human neuroblastoma cell tumorigenesis by inducing p53- and SOX2-mediated cell differentiation. Mol. Carcinog..

[65] Piras S., Furfaro A.L., Brondolo L., Passalacqua M., Marinari U.M., Pronzato M.A., Nitti M. (2017). Differentiation impairs Bach1 dependent HO-1 activation and increases sensitivity to oxidative stress in SH-SY5Y neuroblastoma cells. Sci. Rep..

[66] Fest S., Soldati R., Christiansen N.M., Zenclussen M.L., Kilz J., Berger E., Starke S., Lode H.N., Engel C., Zenclussen A.C. (2016). Targeting of heme oxygenase-1 as a novel immune regulator of neuroblastoma. Int. J. Cancer.

[67] Hassannia B., Wiernicki B., Ingold I., Qu F., Van Herck S., Tyurina Y.Y., Bayır H., Abhari B.A., Angeli J.P.F., Choi S.M. (2018). Nano-targeted induction of dual ferroptotic mechanisms eradicates high-risk neuroblastoma. J. Clin. Investig..

[68] Hayama T., Tabata K., Uchiyama T., Fujimoto Y., Suzuki T. (2011). Ferrearin C induces apoptosis via heme oxygenase-1 (HO-1) induction in neuroblastoma. J. Nat. Med..

[69] Kim B.J., Ryu S.W., Song B.J. (2006). JNK- and p38 kinase-mediated phosphorylation of Bax leads to its activation and mitochondrial translocation and to apoptosis of human hepatoma HepG2 cells. J. Biol. Chem..

[70] Kim H.P., Wang X., Nakao A., Kim S.I., Murase N., Choi M.E., Ryter S.W., Choi A.M. (2005). Caveolin-1 expression by means of p38beta mitogen-activated protein kinase mediates the antiproliferative effect of carbon monoxide. Proc. Natl. Acad. Sci. USA.

[71] Kitano T., Yoda H., Tabata K., Miura M., Toriyama M., Motohashi S., Suzuki T. (2012). Vitamin K3 analogs induce selective tumor cytotoxicity in neuroblastoma. Biol. Pharm. Bull..

[72] Aref D., Croul S. (2013). Medulloblastoma: Recurrence and metastasis. CNS Oncol..

[73] Lamont J.M., McManamy C.S., Pearson A.D., Clifford S.C., Ellison D.W. (2004). Combined histopathological and molecular cytogenetic stratification of medulloblastoma patients. Clin. Cancer Res..

[74] Kool M., Korshunov A., Remke M., Jones D.T., Schlanstein M., Northcott P.A., Cho Y.J., Koster J., Schouten-van Meeteren A., van Vuurden D. (2012). Molecular subgroups of medulloblastoma: An international meta-analysis of transcriptome, genetic aberrations, and clinical data of WNT, SHH, Group 3, and Group 4 medulloblastomas. Acta Neuropathol..

[75] Millard N.E., De Braganca K.C. (2016). Medulloblastoma. J. Child Neurol..

[76] Griffin C.A., Hawkins A.L., Packer R.J., Rorke L.B., Emanuel B.S. (1988). Chromosome abnormalities in pediatric brain tumors. Cancer Res..

[77] Rossi A., Caracciolo V., Russo G., Reiss K., Giordano A. (2008). Medulloblastoma: From molecular pathology to therapy. Clin. Cancer Res..

[78] Kortmann R.D., Kühl J., Timmermann B., Mittler U., Urban C., Budach V., Richter E., Willich N., Flentje M., Berthold F. (2000). Postoperative neoadjuvant chemotherapy before radiotherapy as compared to immediate radiotherapy followed by maintenance chemotherapy in the treatment of medulloblastoma in childhood: Results of the German prospective randomized trial HIT ‘91. Int. J. Radiat. Oncol. Biol. Phys..

[79] Matsutani M. (2004). Chemoradiotherapy for brain tumors: Current status and perspectives. Int. J. Clin. Oncol..

[80] Li T., Yu L. (2014). Clinicopathological significance of HO-1 and HO-2 expression in medulloblastoma. Adv. Mater. Res..

[81] Al-Owais M.M., Scragg J.L., Dallas M.L., Boycott H.E., Warburton P., Chakrabarty A., Boyle J.P., Peers C. (2012). Carbon monoxide mediates the anti-apoptotic effects of heme oxygenase-1 in medulloblastoma DAOY cells via K+ channel inhibition. J. Biol. Chem..

[82] Al-Owais M.M., Dallas M.L., Boyle J.P., Scragg J.L., Peers C. (2015). Heme Oxygenase-1 Influences Apoptosis via CO-mediated Inhibition of K+ Channels. Adv. Exp. Med. Biol..

[83] Buerki R.A., Horbinski C.M., Kruser T., Horowitz P.M., James C.D., Lukas R.V. (2018). An overview of meningiomas. Future Oncol..

[84] Dumanski J.P., Carlbom E., Collins V.P., Nordenskjöld M. (1987). Deletion mapping of a locus on human chromosome 22 involved in the oncogenesis of meningioma. Proc. Natl. Acad. Sci. USA.

[85] Ruttledge M.H., Sarrazin J., Rangaratnam S., Phelan C.M., Twist E., Merel P., Delattre O., Thomas G., Nordenskjöld M., Collins V.P. (1994). Evidence for the complete inactivation of the NF2 gene in the majority of sporadic meningiomas. Nat. Genet..

[86] Rouleau G.A., Merel P., Lutchman M., Sanson M., Zucman J., Marineau C., Hoang-Xuan K., Demczuk S., Desmaze C., Plougastel B. (1993). Alteration in a new gene encoding a putative membrane-organizing protein causes neuro-fibromatosis type 2. Nature.

[87] Clark V.E., Erson-Omay E.Z., Serin A., Yin J., Cotney J., Ozduman K., Avşar T., Li J., Murray P.B., Henegariu O. (2013). Genomic analysis of non-NF2 meningiomas reveals mutations in TRAF7, KLF4, AKT1, and SMO. Science.

[88] Pereira B.J.A., Oba-Shinjo S.M., de Almeida A.N., Marie S.K.N. (2019). Molecular alterations in meningiomas: Literature review. Clin. Neurol. Neurosurg..

[89] Abedalthagafi M., Bi W.L., Aizer A.A., Merrill P.H., Brewster R., Agarwalla P.K., Listewnik M.L., Dias-Santagata D., Thorner A.R., Van Hummelen P. (2016). Oncogenic PI3K mutations are as common as AKT1 and SMO mutations in meningioma. Neuro Oncol..

[90] Simpson D. (1957). The recurrence of intracranial meningiomas after surgical treatment. J. Neurol. Neurosurg. Psychiatry.

[91] Euskirchen P., Peyre M. (2018). Management of meningioma. Presse Med..

[92] Takahashi T., Suzuki S., Misawa S., Akimoto J., Shinoda Y., Fujiwara Y. (2018). Photodynamic therapy using talaporfin sodium induces heme oxygenase-1 expression in rat malignant meningioma KMY-J cells. J. Toxicol. Sci..

[93] Wessels P.H., Twijnstra A., Kessels A.G., Krijne-Kubat B., Theunissen P.H., Ummelen M.I., Ramaekers F.C., Hopman A.H. (2002). Gain of chromosome 7, as detected by in situ hybridization, strongly correlates with shorter survival in astrocytoma grade 2. Genes Chromosomes Cancer.

[94] Reifenberger G., Collins V.P. (2004). Pathology and molecular genetics of astrocytic gliomas. J. Mol. Med..

[95] Ichimura K., Bolin M.B., Goike H.M., Schmidt E.E., Moshref A., Collins V.P. (2000). Deregulation of the p14ARF/MDM2/p53 pathway is a prerequisite for human astrocytic gliomas with G1-S transition control gene abnormalities. Cancer Res..

[96] Ruas M., Peters G. (1998). The p16INK4a/CDKN2A tumor suppressor and its relatives. Biochim. Biophys. Acta.

[97] Hermanson M., Funa K., Hartman M., Claesson-Welsh L., Heldin C.H., Westermark B., Nistér M. (1992). Platelet-derived growth factor and its receptors in human glioma tissue: Expression of messenger RNA and protein suggests the presence of autocrine and paracrine loops. Cancer Res..

[98] Hermanson M., Funa K., Koopmann J., Maintz D., Waha A., Westermark B., Heldin C.H., Wiestler O.D., Louis D.N., von Deimling A. (1996). Association of loss of heterozygosity on chromosome 17p with high platelet-derived growth factor alpha receptor expression in human malignant gliomas. Cancer Res..

[99] Deininger M.H., Meyermann R., Trautmann K., Duffner F., Grote E.H., Wickboldt J., Schluesener H.J. (2000). Heme oxygenase (HO)-1 expressing macrophages/microglial cells accumulate during oligodendroglioma progression. Brain Res..

[100] Hara E., Takahashi K., Tominaga T., Kumabe T., Kayama T., Suzuki H., Fujita H., Yoshimoto T., Shirato K., Shibahara S. (1996). Expression of heme oxygenase and inducible nitric oxide synthase mRNA in human brain tumors. Biochem. Biophys. Res. Commun..

[101] Gandini N.A., Fermento M.E., Salomón D.G., Obiol D.J., Andrés N.C., Zenklusen J.C., Arevalo J., Blasco J., López Romero A., Facchinetti M.M. (2014). Heme oxygenase-1 expression in human gliomas and its correlation with poor prognosis in patients with astrocytoma. Tumour Biol..

[102] Wesseling P., van den Bent M., Perry A. (2015). Oligodendroglioma: Pathology, molecular mechanisms and markers. Acta Neuropathol..

[103] Van den Bent M.J., Chang S.M. (2018). Grade II and III Oligodendroglioma and Astrocytoma. Neurol. Clin..

[104] Griffin C.A., Burger P., Morsberger L., Yonescu R., Swierczynski S., Weingart J.D., Murphy K.M. (2006). Identification of der(1;19)(q10;p10) in five oligodendrogliomas suggests mechanism of concurrent 1p and 19q loss. J. Neuropathol. Exp. Neurol..

[105] Michotte A., Chaskis C., Sadones J., Veld P.I., Neyns B. (2009). Primary leptomeningeal anaplastic oligodendroglioma with a 1p36-19q13 deletion: Report of a unique case successfully treated with Temozolomide. J. Neurol. Sci..

[106] Yan H., Parsons D.W., Jin G., McLendon R., Rasheed B.A., Yuan W., Kos I., Batinic-Haberle I., Jones S., Riggins G.J. (2009). IDH1 and IDH2 mutations in gliomas. N. Engl. J. Med..

[107] Kuroki M., Voest E.E., Amano S., Beerepoot L.V., Takashima S., Tolentino M., Kim R.Y., Rohan R.M., Colby K.A., Yeo K.T. (1996). Reactive oxygen intermediates increase vascular endothelial growth factor expression in vitro and in vivo. J. Clin. Investig..

[108] Furnari F.B., Fenton T., Bachoo R.M., Mukasa A., Stommel J.M., Stegh A., Hahn W.C., Ligon K.L., Louis D.N., Brennan C. (2007). Malignant astrocytic glioma: Genetics, biology, and paths to treatment. Genes Dev..

[109] Wen P.Y., Kesari S. (2008). Malignant gliomas in adults. N. Engl. J. Med..

[110] Kim D.H., Mohapatra G., Bollen A., Waldman F.M., Feuerstein B.G. (1995). Chromosomal abnormalities in glioblastoma multiforme tumors and glioma cell lines detected by comparative genomic hybridization. Int. J. Cancer.

[111] Rasheed B.K., McLendon R.E., Friedman H.S., Friedman A.H., Fuchs H.E., Bigner D.D., Bigner S.H. (1995). Chromosome 10 deletion mapping in human gliomas: A common deletion region in 10q25. Oncogene.

[112] Shinojima N., Tada K., Shiraishi S., Kamiryo T., Kochi M., Nakamura H., Makino K., Saya H., Hirano H., Kuratsu J. (2003). Prognostic value of epidermal growth factor receptor in patients with glioblastoma multiforme. Cancer Res..

[113] Fulci G., Labuhn M., Maier D., Lachat Y., Hausmann O., Hegi M.E., Janzer R.C., Merlo A., Van Meir E.G. (2000). p53 gene mutation and ink4a-arf deletion appear to be two mutually exclusive events in human glioblastoma. Oncogene.

[114] Ishii N., Maier D., Merlo A., Tada M., Sawamura Y., Diserens A.C., Van Meir E.G. (1999). Frequent co-alterations of TP53, p16/CDKN2A, p14ARF, PTEN tumor suppressor genes in human glioma cell lines. Brain Pathol..

[115] Zhu J., Wang H., Sun Q., Ji X., Zhu L., Cong Z., Zhou Y., Liu H., Zhou M. (2013). Nrf2 is required to maintain the self-renewal of glioma stem cells. BMC Cancer.

[116] Sun X., Ou Z., Chen R., Niu X., Chen D., Kang R., Tang D. (2016). Activation of the p62-Keap1-NRF2 pathway protects against ferroptosis in hepatocellular carcinoma cells. Hepatology.

[117] Zhu J., Wang H., Fan Y., Hu Y., Ji X., Sun Q., Liu H. (2014). Knockdown of nuclear factor erythroid 2-related factor 2 by lentivirus induces differentiation of glioma stem-like cells. Oncol. Rep..

[118] Cisowski J., Loboda A., Józkowicz A., Chen S., Agarwal A., Dulak J. (2005). Role of heme oxygenase-1 in hydrogen peroxide-induced VEGF synthesis: Effect of HO-1 knockout. Biochem. Biophys. Res. Commun..

[119] Dey S., Sayers C.M., Verginadis I.I., Lehman S.L., Cheng Y., Cerniglia G.J., Tuttle S.W., Feldman M.D., Zhang P.J., Fuchs S.Y. (2015). ATF4-dependent induction of heme oxygenase 1 prevents anoikis and promotes metastasis. J. Clin. Investig..

[120] Ghosh D., Ulasov I.V., Chen L., Harkins L.E., Wallenborg K., Hothi P., Rostad S., Hood L., Cobbs C.S. (2016). TGFβ-Responsive HMOX1 Expression Is Associated with Stemness and Invasion in Glioblastoma Multiforme. Stem Cells.

[121] Baird L., Dinkova-Kostova A.T. (2011). The cytoprotective role of the Keap1-Nrf2 pathway. Arch Toxicol..

[122] Cong Z.X., Wang H.D., Wang J.W., Zhou Y., Pan H., Zhang D.D., Zhu L. (2013). ERK and PI3K signaling cascades induce Nrf2 activation and regulate cell viability partly through Nrf2 in human glioblastoma cells. Oncol. Rep..

[123] Pan H., Wang H., Zhu L., Wang X., Cong Z., Sun K., Fan Y. (2013). The involvement of Nrf2-ARE pathway in regulation of apoptosis in human glioblastoma cell U251. Neurol. Res..

[124] Liu Y., Liang Y., Zheng T., Yang G., Zhang X., Sun Z., Shi C., Zhao S. (2011). Inhibition of heme oxygenase-1 enhances anti-cancer effects of arsenic trioxide on glioma cells. J. Neurooncol..

[125] Zhang L., Wang H. (2017). FTY720 inhibits the Nrf2/ARE pathway in human glioblastoma cell lines and sensitizes glioblastoma cells to temozolomide. Pharmacol. Rep..

[126] El Andaloussi A., Lesniak M.S. (2007). CD4+ CD25+ FoxP3+ T-cell infiltration and heme oxygenase-1 expression correlate with tumor grade in human gliomas. J. Neurooncol..

[127] Kim S.S., Seong S., Lim S.H., Kim S.Y. (2013). Biliverdin reductase plays a crucial role in hypoxia-induced chemoresistance in human glioblastoma. Biochem. Biophys. Res. Commun..

[128] Kwon M.Y., Park E., Lee S.J., Chung S.W. (2015). Heme oxygenase-1 accelerates erastin-induced ferroptotic cell death. Oncotarget.

[129] Lu B., Chen X.B., Ying M.D., He Q.J., Cao J., Yang B. (2017). The Role of Ferroptosis in Cancer Development and Treatment Response. Front. Pharmacol..

[130] Dixon S.J., Lemberg K.M., Lamprecht M.R., Skouta R., Zaitsev E.M., Gleason C.E., Patel D.N., Bauer A.J., Cantley A.M., Yang W.S. (2012). Ferroptosis: An iron-dependent form of nonapoptotic cell death. Cell.

[131] Kyani A., Tamura S., Yang S., Shergalis A., Samanta S., Kuang Y., Ljungman M., Neamati N. (2018). Discovery and Mechanistic Elucidation of a Class of Protein Disulfide Isomerase Inhibitors for the Treatment of Glioblastoma. ChemMedChem.

[132] Podkalicka P., Mucha O., Jozkowicz A., Dulak J., Loboda A. (2018). Heme oxygenase inhibition in cancers: Possible tools and targets. Contemp. Oncol..

[133] Sorrenti V., Guccione S., Di Giacomo C., Modica M.N., Pittala V., Acquaviva R., Basile L., Pappalardo M., Salerno L. (2012). Evaluation of imidazole-based compounds as heme oxygenase-1 inhibitors. Chem. Biol. Drug Des..

[134] Salerno L., Amata E., Romeo G., Marrazzo A., Prezzavento O., Floresta G., Sorrenti V., Barbagallo I., Rescifina A., Pittala V. (2018). Potholing of the hydrophobic heme oxygenase-1 western region for the search of potent and selective imidazole-based inhibitors. Eur. J. Med. Chem..

[135] Greish K.F., Salerno L., Al Zahrani R., Amata E., Modica M.N., Romeo G., Marrazzo A., Prezzavento O., Sorrenti V., Rescifina A. (2018). Novel Structural Insight into Inhibitors of Heme Oxygenase-1 (HO-1) by New Imidazole-Based Compounds: Biochemical and In Vitro Anticancer Activity Evaluation. Molecules.

[136] Sorrenti V., Pittala V., Romeo G., Amata E., Dichiara M., Marrazzo A., Turnaturi R., Prezzavento O., Barbagallo I., Vanella L. (2018). Targeting heme Oxygenase-1 with hybrid compounds to overcome Imatinib resistance in chronic myeloid leukemia cell lines. Eur. J. Med Chem..

[137] Ciaffaglione V., Intagliata S., Pittala V., Marrazzo A., Sorrenti V., Vanella L., Rescifina A., Floresta G., Sultan A., Greish K. (2020). New Arylethanolimidazole Derivatives as HO-1 Inhibitors with Cytotoxicity against MCF-7 Breast Cancer Cells. Int. J. Mol. Sci..

[138] Raffaele M., Pittala V., Zingales V., Barbagallo I., Salerno L., Li Volti G., Romeo G., Carota G., Sorrenti V., Vanella L. (2019). Heme Oxygenase-1 Inhibition Sensitizes Human Prostate Cancer Cells towards Glucose Deprivation and Metformin-Mediated Cell Death. Int. J. Mol. Sci..

